# Vascular Normalization Induced by Sinomenine Hydrochloride Results in Suppressed Mammary Tumor Growth and Metastasis

**DOI:** 10.1038/srep08888

**Published:** 2015-03-09

**Authors:** Huimin Zhang, Yu Ren, Xiaojiang Tang, Ke Wang, Yang Liu, Li Zhang, Xiao Li, Peijun Liu, Changqi Zhao, Jianjun He

**Affiliations:** 1 Department of Surgical Oncology, The First Affiliated Hospital of Xi'an Jiaotong University, 277 West Yanta Road, Xi'an 710061, P.R. China; 2 Translational Medical center, The First Affiliated Hospital of Xi'an Jiaotong University, 277 West Yanta Road, Xi'an 710061, P.R. China; 3 Key Laboratory of Cell Proliferation and Regulation Biology, College of Life Science, Beijing Normal University, Beijing 100875, P.R. China

## Abstract

Solid tumor vasculature is characterized by structural and functional abnormality and results in a hostile tumor microenvironment that mediates several deleterious aspects of tumor behavior. Sinomenine is an alkaloid extracted from the Chinese medicinal plant, *Sinomenium acutum*, which has been utilized to treat rheumatism in China for over 2000 years. Though sinomenine has been demonstrated to mediate a wide range of pharmacological actions, few studies have focused on its effect on tumor vasculature. We showed here that intraperitoneally administration of 100 mg/kg sinomenine hydrochloride (SH, the hydrochloride chemical form of sinomenine) in two orthotopic mouse breast cancer models for 14 days, delayed mammary tumor growth and decreased metastasis by inducing vascular maturity and enhancing tumor perfusion, while improving chemotherapy and tumor immunity. The effects of SH on tumor vessels were caused in part by its capability to restore the balance between pro-angiogenic factor (bFGF) and anti-angiogenic factor (PF4). However 200 mg/kg SH didn't exhibit the similar inhibitory effect on tumor progression due to the immunosuppressive microenvironment caused by excessive vessel pruning, G-CSF upregulation, and GM-CSF downregulation. Altogether, our findings suggest that SH induced vasculature normalization contributes to its anti-tumor and anti-metastasis effect on breast cancer at certain dosage.

Breast cancer is the most common malignant tumor as well as the leading cause of death from cancer among women globally^1^. Currently, the chemotherapy for breast cancer is underdeveloped especially in the aggressive type with a high risk of recurrence or metastasis although there are some targeted drugs or combined medication scheme being utilized. Therefore, it is urgent to develop new therapeutic approaches that are feasible, affordable and less toxic for control of both the actual tumor load and the metastatic potential of the disease.

Pathological angiogenesis — driven by an imbalance between pro- and anti-angiogenic signaling — is one of the hallmarks of cancers, including breast cancer^2^. Unlike blood vessels in normal tissues, blood vessel growth in tumor is not only stimulated, but these vessels are also structurally and functionally abnormal^3^. The pathological angiogenesis results in a hostile tumor microenvironment characterized by interstitial hypertension, hypoxia and acidosis that can alter the intrinsic characteristics of tumor cells so malignant tumor clones with increased metastatic potential are selected and escape of tumor cells through leaky vessels is facilitated^3^,^4^. The irregular, disorderly structured and inefficiently perfused tumor vessels can also impede immune cell proliferation, infiltration, survival and function in tumor^5^. As a result, the abnormal tumor vasculature can also lead to resistance of tumor cells to radiation therapy and many chemotherapeutics^6^,^7^. In addition, hypoxia upregulates the production of angiogenic factors, further aggravate vessel disorganization and thereby fuel non-productive angiogenesis in an endless self-reinforcing loop^4^. Traditional antiangiogenic “vessel pruning” agents can aggravate tumor hypoxia and worsen malignancy^8^. Antiangiogenic “vessel normalizing” strategies are gaining attention, as they can reduce metastasis and improve anticancer therapies^9^.

The Chinese medicinal herb *Sinomenium acutum* has been successfully used for centuries in the treatment of rheumatism and neuralgia with extremely low side effect in many areas of the Far East^10^. Sinomenine hydrochloride (SH) is the hydrochloride chemical form of sinomenine, a pure alkaloid and the active principle extracted from this herb. It has previously been demonstrated that SH exhibited a wide range of pharmacological actions including anti-inflammatory, immunoregulatory, anti-angiogenic, anti-arrhythmic, as well as mild sedative and analgesic effects^10^,^11^,^12^,^13^. Recently, a number of studies have reported the anti-proliferation potential of SH against a variety of human tumor cells by inducing apoptosis and cell cycle arrest, including hepatocellular carcinoma, lung cancer, synovial sarcoma and gastric cancer cells^14^,^15^,^16^,^17^.

Despite significant efforts in anti-cancer researches with SH, little attention has been paid to the tumor vascular system after SH treatment which may have great effect on the tumor microenvironment. This study investigated whether SH could inhibit breast tumor growth or metastasis through tumor vasculature changes. The results demonstrated that 100 mg/kg SH was effective for suppressing 4T1 and 168FARN tumors growth and 4T1 cells spreading through inducing vessel normalization, while 200 mg/kg SH had no inhibition effect on tumor progression because of the excessive vessel pruning and change of immune status.

## Results

### SH interferes with endothelial cell functions without causing endothelial cell death

**Figure 1:**
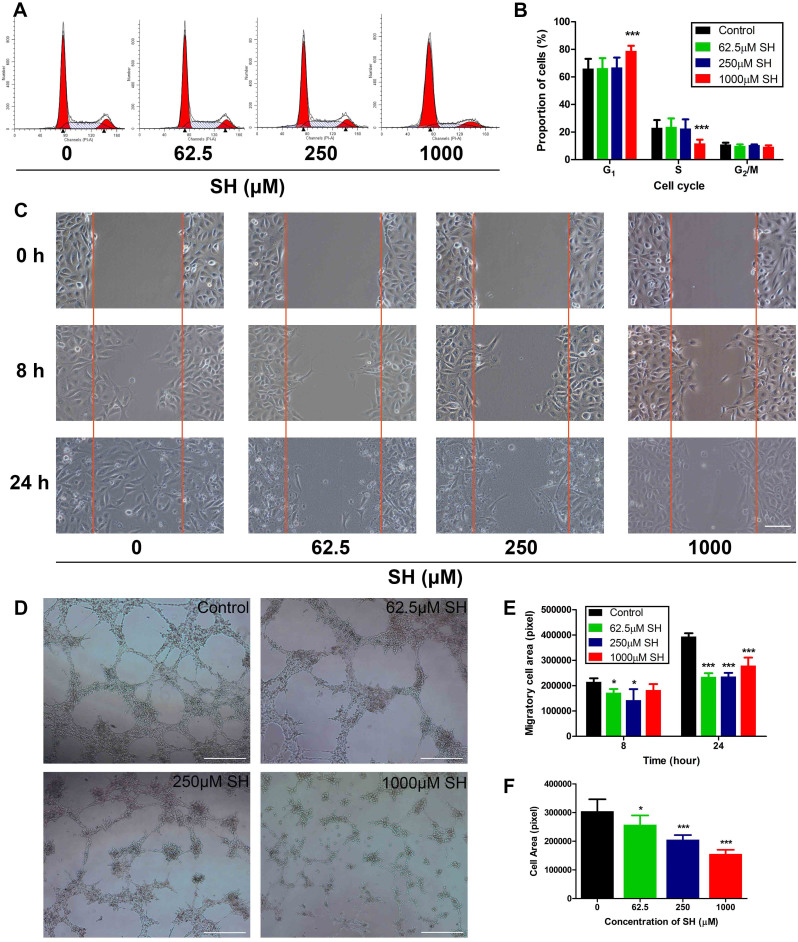
SH interferes with endothelial cell function without causing endothelial cell death. (A, B) Flow cytometric analysis of HUVECs cell cycle treated with SH for 48 hours, qualified in (B). (C, E) HUVECs migration was demonstrated by wound healing assay after SH treatment, qualified in (E). Bars: 200 μm. (D, F) The capability of HUVECs to form capillary-like network was observed after 24 hours SH pre-treatment, qualified in (F). Bars: 1000 μm. Statistical significance: P < 0.05 (*) or P < 0.001 (***), N = 3.

At a concentration ranging from 31.25 μM to 1000 μM, SH showed mild cytotoxicity on HUVECs during 72 hours (Supplementary Fig. S1). With 1 mM SH for 48 h, HUVECs were found to accumulate in G1 phase, whereas the number of cells in S phase decreased significantly after incubation (Fig. 1A, B). HUVECs migration was suppressed by SH even at very low concentration (Fig. 1C, E). Since endothelial cell proliferation and migration are essential steps involved in the process of angiogenesis, we further demonstrated the anti-angiogenic activities of SH by the tube formation assay. Compared with medium control, SH-treated HUVECs formed incomplete tube-like structures (Figure 1D) and the extent of tube formation of HUVEC was reduced significantly in a SH-dose-dependent manner (Figure 1F).

### Effects of SH on primary tumor growth and spontaneous metastasis in vivo

**Figure 2:**
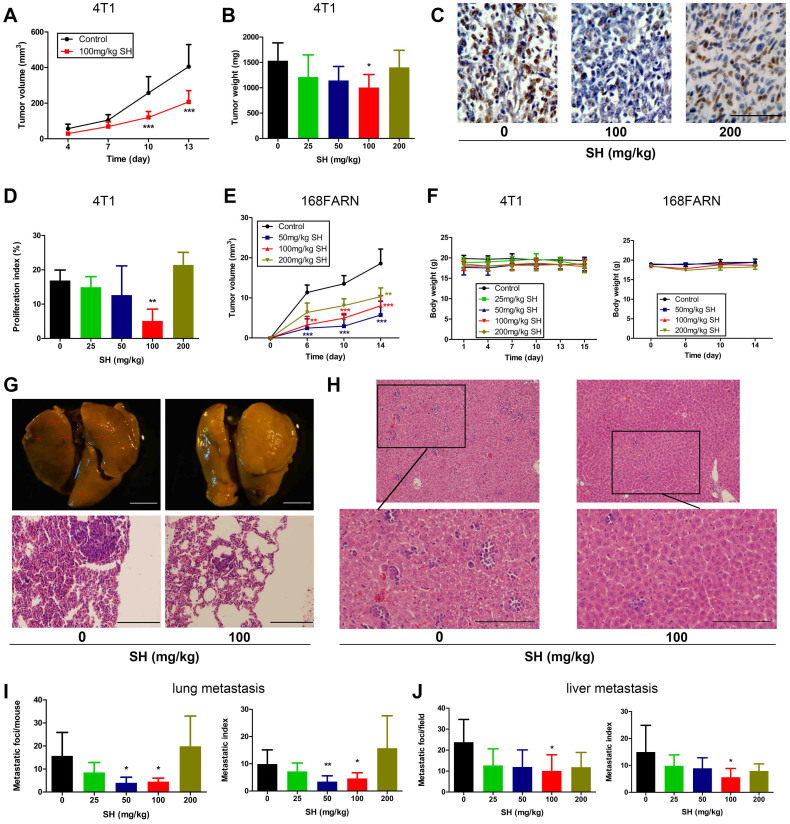
Effect of SH on primary tumor growth and spontaneous metastasis in vivo. (A) Primary tumor volumes were determined regularly in 4T1 tumor models. (B) Tumor burdens after 14-day treatment by different dose of SH were measured. (C) PCNA staining was carried out in control and SH-treated tumors. Bars: 100 μm; (D) proliferation index (PCNA^+^/total cells). (E) Tumor volumes of 168FARN tumor models were measured every 4 days. (F) Body weights throughout the treatment were determined in both models. (G) Visualized lung metastasis in 4T1 tumor models after resection (arrows: metastatic foci). Bars: 2.5 mm; HE staining of lung sections. Bars: 200 μm. (H) HE staining of liver sections showing tumor hepatic metastasis in 4T1 tumor models. Bars: 200 μm. (I, J) The number of metastatic foci was counted and metastatic index was determined as nodules per gram tumor. Statistical significance: P < 0.05 (*), P < 0.01 (**) or P < 0.001 (***), N = 6.

We next examined the effects of SH on inhibition of a 4T1 murine breast cancer model using different doses of SH. The growth of primary tumors in mice treated with 100 mg/kg SH was decreased compared with that of tumors in control group (Fig. 2A). After 14 days of treatment, only the dose of 100 mg/kg caused significant reduction in tumor weight by 31% compared with control (Fig. 2B). Consistent with the reduction in tumor growth, PCNA staining was also decreased in 100 mg/kg SH-treated tumors (Fig. 2C, D). Since this tumor is an animal model for stage IV human breast cancer, we then used another murine breast cancer model to mimic the early stage tumor. 168FARN cells which are less aggressive and could only spread to the regional lymph nodes through lymphatic circulation were injected at 2 × 10^5^ into the mammary fat pad. Result showed that lower dose of SH exhibited stronger inhibition on tumor growth (Fig. 2E). We did not find any gross signs of toxicity following SH administration in either model, demonstrated by no obvious changes in body weight throughout the study (Fig. 2F).

Meanwhile, 100 mg/kg SH also decreased 4T1 lung metastasis (Fig. 2G, I) and liver metastasis (Fig. 2H, J) by 71% and 55% respectively, indicating 100 mg/kg SH to be the optimal beneficial dose in the model studied. As SH decreased the metastatic index (nodules per gram tumor) (Fig. 2I, J), the reduced tumor spread was partly independent of tumor growth inhibition. Since SH had virtually no cytotoxic effects on these cells in vitro (IC_50_ > 1000 μM for 24 h treatment, > 500 μM for 48 h treatment and >300 μM for 72 h treatment) (Supplementary Fig. S2), stromal rather than tumor cell autonomous mechanisms accounted for the decreased tumor cell proliferation in vivo. We thus focused on vasculature, known to regulate tumor growth and metastasis.

### SH inhibits angiogenesis and promotes vessel normalization

**Figure 3:**
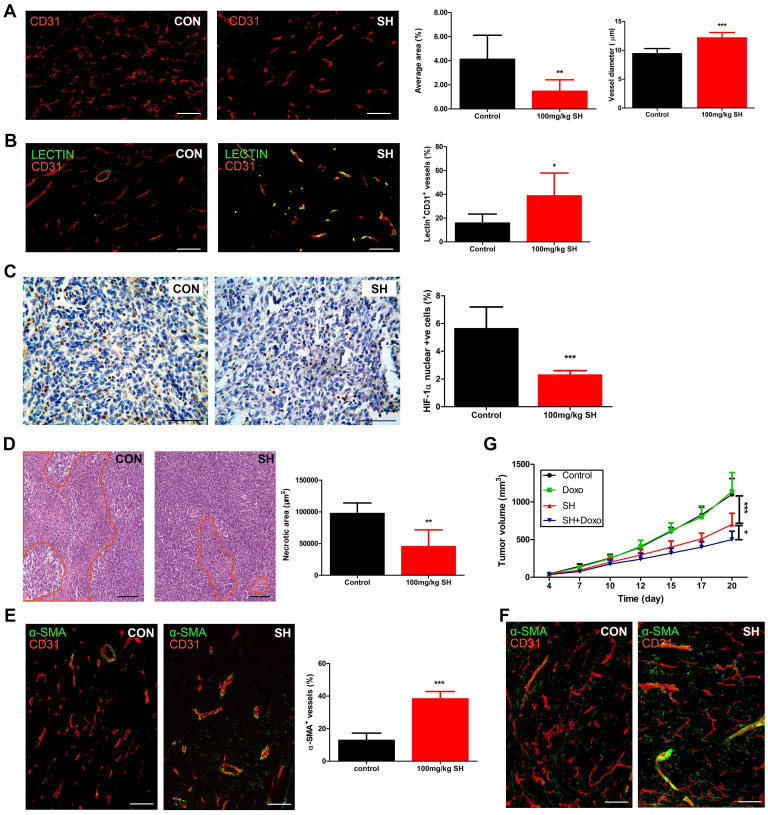
SH inhibits angiogenesis and promotes vessel normalization. (A) Staining for CD31 (red) showing the vessel area (CD31^+^ area, %) and vessel diameters in tumors. (B) Staining for FITC-conjugated lectin (green) and CD31 (red) indicating perfused lectin^+^CD31^+^ vessels (% of CD31^+^ vessels) in tumors. (C) The tumor oxygen supply was determined by HIF-1α staining (HIF-1α^+^/total cells). (D) HE staining showing necrosis (necrotic area is marked with red lines) in tumors. (E) Double staining for CD31 (red) and α-SMA (green) showing pericyte-covered tumor vessels within tumors which is further emphasized by confocal microscopy analysis of double staining of thick tumor sections followed by 3D projection of z-slice images (F). (G) Tumor volumes in mice injected intravenously with a suboptimal dose of doxorubicin (Doxo) or saline combined with SH administration. Bars: 100 μm. Statistical significance: P < 0.05 (*), P < 0.01 (**) or P < 0.001 (***), N = 6–8.

Staining for the endothelial cell marker CD31 showed that not only the average vessel area was reduced significantly after SH treatment, the vessel morphology was also changed (Fig. 3A). Tumor vessels from control group followed serpentine course with irregular and heterogeneous structures. In contrast, vessels in SH-treated mice appeared less tortuous and more organized. Besides, SH-treated tumors also showed an increase in vessel diameter. Since these parameters do not necessarily correlate with vessel function, perfusion was studied by delivery of FITC-conjugated lectin. Double the number of vessels was perfused in SH-treated tumors (Fig. 3B). Meanwhile tumors were less hypoxic, as assessed by staining (Fig. 3C) and western blot (Supplementary Fig. S3) of HIF-1α whose accumulation and activity is precisely regulated by the cellular O_2_ concentration, and displayed smaller necrotic areas (Fig. 3D). Since pericyte coverage improves vessel maturation, we double stained for CD31 and the pericyte marker α-smooth muscle actin (α-SMA) and observed an increased pericyte coverage of tumor vessels in SH-treated tumor (Fig. 3E). Furthermore, confocal microscopy analysis of double staining of thick tumor sections followed by 3D projection of z-slice images emphasized the impact of SH on tumor vessel architecture and mural cell package (Fig. 3F).

Given the observed vessel normalization and reduction in hypoxia, we then studied the effect of SH on chemotherapy sensitivity. Intravenous administration of doxorubicin at a dose of 2.5 mg/kg, 3×/wk was ineffective in reducing the growth of control tumor, but decreased SH-treated tumor growth by 50% (Fig. 3G).

### Effect of SH on metastatic tumor cell extravasation into the lungs

**Figure 4:**
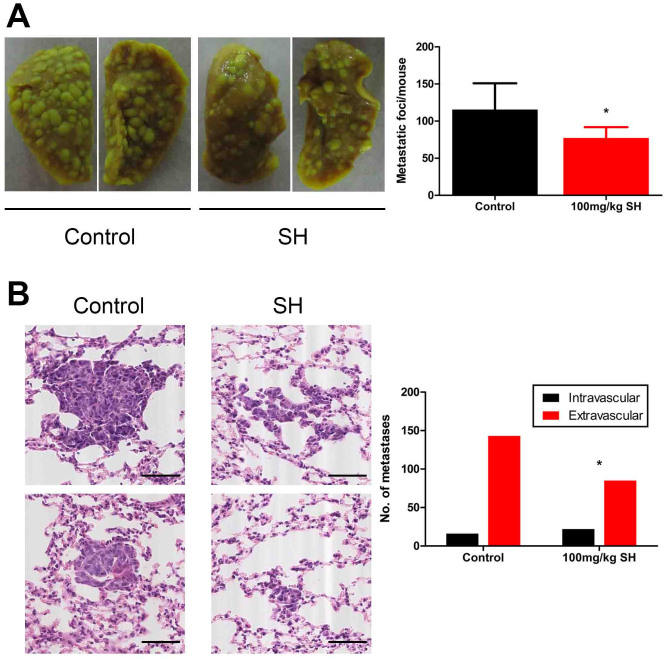
Effect of SH on extravasation of disseminated tumor cells into distant organ parenchyma. (A) Representative pictures of visualized metastasis on lung surface. (B) Representative HE stained sections of micrometastases. Control lungs (left panels) show micrometastatic cells that have breached vascular boundaries and entered the alveolar airspace. SH–treated lungs (right panels) show cells tracking within vessels yet to extravasate. Number of intra- vs extravascular micrometastases was counted. Bars: 100 μm. Statistical significance: P < 0.05 (*), N = 5.

To determine if the effect of SH on metastasis was specifically due to improved vessel normalization, we used an experimental metastasis model. 4T1 cells, known to have an extended intravascular growth phase after homing to the lungs, were intravenously injected, and SH treatment was commenced 12 hours later. After 9 days, the lungs of SH-treated mice showed fewer macroscopic metastatic nodules (Fig. 4A). Histologically, SH-treated mice showed more clumps of metastatic cells lodged within alveolar vessels that failed to breach the vascular basement membrane and to enter the lung parenchyma (Fig. 4B).

### SH modulates angiogenesis-related cytokines in tumors

**Figure 5:**
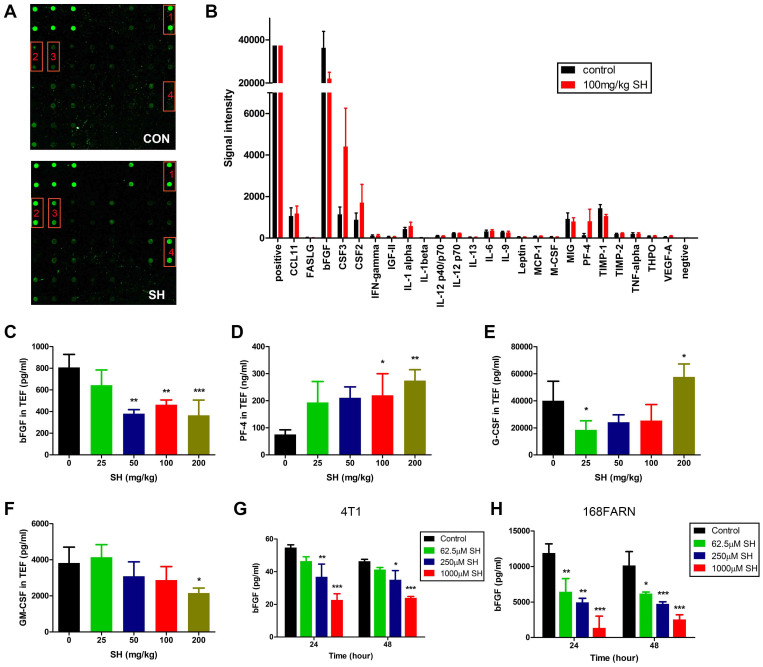
Modulation effect of SH on intratumoral angiogenic factors. (A) Representive pictures of angiogenesis antibody array (1: bFGF, 2: CSF3, 3: CSF2, 4: PF-4) from control and SH-treated group, quantified in B (signal intensity of each spot). N = 4. (C–F) Changes of secreted bFGF, PF-4, G-CSF and GM-CSF in tumor extracellular fluid were measured by ELISA assay. N = 5. (G–H) bFGF levels in cell lysate were measured by ELISA assay. N = 3. Statistical significance: P < 0.05 (*), P < 0.01 (**) or P < 0.001 (***).

To uncover the mechanism through which SH induces vessel normalization, expression of a set of proteins known to regulate angiogenesis, inflammation and apoptosis was analyzed using an antibody array on whole-protein extracts of tumors. Of all 24 candidates analyzed, levels of granulocyte-macrophage colony-stimulating factor (GM-CSF, CSF2), granulocyte colony-stimulating factor (G-CSF, CSF3) and platelet factor-4 (PF-4) were increased 1.9-fold, 3.9-fold and 6.4-fold respectively by SH treatment, while expression of one of the most important pro-angiogenic factors, basic fibroblast growth factor (bFGF), was decreased by 40% (Fig. 5A, B). We next examined the level of bFGF, PF-4, G-CSF and GM-CSF in tumor extracellular fluid in different dose groups to verify the results. bFGF was downregulated in several doses (Fig. 5C) while PF-4 was upregulated (Fig. 5D). Interestingly the level of secreted GM-CSF and G-CSF was unaffected in 100 mg/kg group, but was significantly influenced in 200 mg/kg group (Fig. 5E, F). Change of G-CSF in serum was not significant (Fig. S4A, B) and GM-CSF was undetectable in serum. To further determine the SH-dependent source of bFGF in tumors, we analyzed its expressions in tumor cells and HUVECs when treated with SH. bFGF levels were decreased in both 4T1 and 168FARN cells (Fig. 5G, H) while bFGF in HUVEC is very low and almost unaffected by SH treatment (Fig. S4C). Altogether, these data indicated that SH could modulate intratumoral angiogenic factor levels.

### Effect of SH on TAMs and MDSCs in tumors

**Figure 6:**
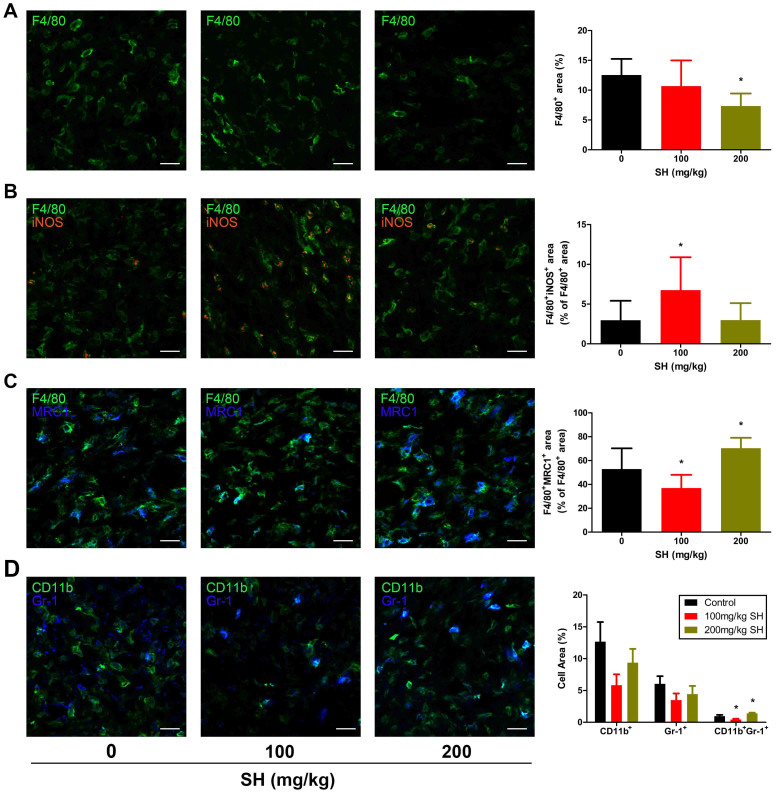
Effect of SH on TAMs and MDSCs. (A) Staining of F4/80, revealing macrophages infiltration within tumors. (B) Double staining for F4/80 (green) and iNOS (red), showing M_1_ TAMs (F4/80^+^iNOS^+^) in tumors. (C) Double staining for F4/80 (green) and MRC1 (blue), indicating M_2_ TAMs (F4/80^+^MRC1^+^) within tumors. (D) Double staining for CD11b (green) and Gr-1 (blue), revealing MDSCs (CD11b^+^Gr-1^+^) accumulation in tumors. Bars: 25 μm. Statistical significance: P < 0.05 (*), N = 6.

As GM-CSF and G-CSF are also important chemotactic factors for tumor associated macrophages (TAMs) and myeloid-derived suppressor cells (MDSCs), we supposed that the significant changes of these two factors may affect the antitumor immunity which could cause the entirely different effects in 200 mg/kg SH-treated group. Immunostaining of F4/80 showed that the macrophages accumulation was significantly reduced in 200 mg/kg group but was unaffected in 100 mg/kg group (Fig. 6A). Next we analyzed the phenotype of TAMs and found that fewer F4/80^+^TAMs expressed MRC1 and more expressed iNOS in 100 mg/kg SH-treated tumors, which indicates fewer protumoral and proangiogenic (M2-like) TAMs and more antitumoral and proinflammatory (M1-like) TAMs existed within these tumors (Fig. 6B,C). Conversely, M2-like TAMs was increased in 200 mg/kg SH-treated tumors. Meanwhile, accumulation of CD11b^+^Gr-1^+^ cells was suppressed in 100 mg/kg group but promoted in 200 mg/kg group (Fig. 6D). In conclusion, 100 mg/kg SH treatment could improve the antitumor immunity by inducing vessel normalization. 200 mg/kg SH treatment could alter this effect by excessive vessel prune (Supplementary Fig. S5) and changes of GM-CSF and G-CSF.

## Discussion

In this study, we report that SH combats tumor progression and dissemination by promoting vessels normalization at certain dosage. Critically underlying these activities is the ability of SH to restore the balance between angiogenesis stimulators and inhibitors.

Untreatable metastasis is often the cause of mortality in cancer patients. A prominent environmental stimulus of tumor dissemination is hypoxia, resulting from poorly functioning abnormalized tumor vessels. Our findings suggest that 100 mg/kg SH inhibited tumor growth and metastasis in part by altering vessel morphology. These changes, ranging from decreased vessel tortuosity, reduced vessel density, increased vessel diameter and increased pericyte coverage, promoted vessel normalization, perfusion and oxygenation. Hence, by creating a less hypoxic milieu, SH diminished the need for tumor cell to escape. Since hypoxia stimulates M2-like polarization^18^, improved oxygenation in 100 mg/kg SH treated tumors can provide a self-reinforcing stimulus for further polarizing TAMs away from an M2-like phenotype to an M1-like phenotype. At the same time, vascular normalizing doses of antiangiogenic treatment reprogram the immunosuppressive tumor microenvironment^5^,^19^, the inhibition effect of 100 mg/kg SH on CD11b^+^Gr-1^+^ MDSCs recruitment is greatly due to the improved tumor perfusion. Tumor hypoxia is also known to confer resistance to various treatment such as radiation, certain chemotherapies, photodynamic therapy and even immunotherapies^20^, so there may be more possibilities for SH to perform its antitumoral effect by combination therapy. Clinical studies also showed that increased tumor blood perfusion caused by vascular normalization is associated with longer survival in cancer patients^21^.

The early phase of tumor growth is most clinically relevant to the progression of micrometastases to macroscopic tumors. Here, we show that when started SH treatment the day after tumor cells were injected into the mammary fat pad, the angiogenesis was blocked and the newly formed vessels were induced to be of a more mature structure. As a result, the metastatic tumor cells to the distant organ's intravascular compartment were constrained; hence the metastatic progression was delayed.

In physiologic angiogenesis, the effects of proangiogenic molecules, such as VEGF and bFGF, are exquisitely counterbalanced by endogenous antiangiogenic molecules, like sVEGFR1 and thrombospondins^3^,^22^. During tumor angiogenesis, the balance is tipped in favor of new vessel formation with abnormal structure and function. So tip the imbalance between pro- and antiangiogenic factors back toward equilibrium by mopping up some of the excessive proangiogenic factors or/and releasing some antiangiogenic factors could normalize tumor vasculature^23^. In this study, we measured angiogenic proteins in tumor lysate and tumor extracellular fluid indicating a regulatory effect of SH on the expression of several angiogenic factors. Remarkably, expression of bFGF, which is very important for almost all steps in the angiogenesis process, like degradation of basement membrane, migration of endothelial cells into interstitial space and sprouting and endothelial cell proliferation^24^, is reduced by 40% in tumors treated with SH. *In vitro* assay demonstrated that tumor cell is the SH-dependent source of bFGF. A reduction of bFGF level will also inhibit antiapoptotic effects of bFGF on tumor cells^25^. Another protein with significant change is PF-4, an antiangiogenic factor, which inhibits endothelial cell proliferation, migration, microvessel assembly, and *in vivo* suppression of new blood vessel formation at micromolar doses by specifically interacting with bFGF by complex formation, inhibiting bFGF dimerization, binding to FGFR-1 and internalization^26^,^27^,^28^. With 6-fold increase of PF-4, SH does not only decrease the expression of bFGF, but also interferes with the bFGF/FGFR function.

Interestingly, when SH was used at higher dose –200 mg/kg, they are likely to prunes tumor vessels excessively, rather than normalizing them, and paradoxically may severe tumor hypoxia and thus compromise various therapies. This excessive pruning also may exacerbate, rather than reverse, the immunosuppressive tumor microenvironment^19^. At the same time, two colony stimulating factors, which are regulated by 200 mg/kg SH, play critical roles in immune microenvironment. High G-CSF production has been associated with a poor prognosis in cancer patients^29^. It promotes tumor refractoriness to anti-angiogenic therapy targeting VEGF-A and angiopoietin-2 by inducing myeloid cell recruitment and rescuing neo-angiogenesis^29^,^30^,^31^. Meanwhile G-CSF produced by tumor and stromal cells is involved in the differentiation of progenitors into CD11b^+^/Gr-1^+^ cells of the myeloid lineage and/or the mobilization of these cells to the peripheral blood^32^. CD11b^+^/Gr-1^+^ cells can facilitate tumor growth by virtue of their ability to downregulate the immune responses in subtypes of T-cells such as CD4^+^ and CD8^+^ cells; hence, the denomination of MDSCs for at least a subset of CD11b^+^/Gr1^+^ cells^33^. Multiple growth factors including bFGF could stimulate G-CSF expression by activate RAS signaling pathway through MAPK-induced Ets2 transcriptional activity^29^,^34^. Here, we found G-CSF expression was increased rather than decreasing with bFGF. Further study is needed to uncover the mechanism under this phenomenon. It is demonstrated that GM-CSF treatment of breast cancer slows tumor growth and metastasis by stimulating monocytes to express and release sVEGFR-1 which blocks detection of VEGF and inhibits angiogenesis both *in vitro* and *in vivo*^35^,^36^. Meanwhile, injection of irradiated, GM-CSF transfected tumor cells stimulates an intense local inflammatory reaction consisting of DCs, macrophages and granulocytes, indicating that GM-CSF functioned to increase tumor antigen presentation^37^. So the declined GM-CSF in 200 mg/kg SH-treated tumors might contribute to the protumoral effect of SH in this dosage by suppressing anti-tumor immunity.

This is the first time that the totally different effects of 100 mg/kg and 200 mg/kg SH have been reported, indicating the importance of proper dose range when treat cancer with this drug. Here, we used two murine breast cancer models to mimic early and late stage of human breast cancer which were detailed described and widely utilized in breast cancer research^38^. Although the dose dependent effect of SH was investigated in both models, it's necessary to verify the discovery in a series of human breast cancer cell lines for pre-clinical study. We found regulation of angiogenic factors – bFGF and PF4, and chemotactic factors – GM-CSF and G-CSF within tumor microenvironment by SH led to remarkable changes in tumor vasculature and immune status. While based on previous studies, SH does exhibit a wide range of effects through different pathways, if there's other molecular mechanisms play roles in the anti-tumor and anti-metastasis effect on breast cancer need to be further investigated.

In summary, we propose SH, a drug with a long history, exhibits antitumor effect by suppressing angiogenesis and inducing vessel normalization at certain dosage. Unlike cytotoxicity drugs, SH works to stabilize vessels and normalize their function and hence suppresses tumor growth and metastasis, moreover, enhance the chemotherapy sensitivity and improve antitumor immunity. Our data provide novel pharmacological insights into the therapeutic effect of SH on tumor progression and dissemination.

## Methods

### Cell proliferation and cell cycle analysis

Sinomenine hydrochloride (SH) was purchased from Shaanxi Baoji Yongjia Natural Plant Development Co., Ltd and the purity of the drug was confirmed by reverse phase-HPLC (>99.8%). Cells were seeded at 2000/well in 96-well plates and MTT solution was added to each well and incubated for 4 h at certain time points. After adding DMSO, the absorbance at 490 nm was measured with the Multimode Reader (EnSpire PerkinElmer, USA). For cell cycle analysis, cultured cells were harvested, washed twice by PBS, and fixed in 70% ethanol at 4°C for 4 h. Cells were incubated with RNase (1 mg/ml) at 37°C for 30 min and propidium iodide (40 μg/ml) at 4°C away from light for 30 min and were analyzed by flow cytometry (BD FACSCanto II, USA).

### Migration assay and tube formation assay

Cell migration was measured using a wound healing assay. Create a wound by manually scraping the cell monolayer formed by HUVECs with a 20 μl pipet tip. Images were captured using inverted microscope with camera attachment (Nikon Eclipse Ti-S) at 0, 8 and 24 h after SH treatment. For tube formation assay, HUVECs were pre-treated with culture medium and different concentrations of SH for 24 h. The pre-treated cells were then seeded at a density of 8 × 10^4^/well on the Matrigel-coated 48-well plate. The overviews of the network-like structures were captured using inverted microscope with camera attachment (Nikon Eclipse Ti-S, Japan) at 6 h.

### In vivo tumorigenesis assay and metastasis assay

Female Balb/c mice (6–8 weeks old, body weight 18–20 g) were purchased from Laboratory Animal Center of Xi'an Jiaotong University (Xi'an, China). All animals were housed at pathogen-free condition. For spontaneous metastasis study, 4T1 cells were harvested and single-cell suspensions of 1 × 10^6^ cells in 100 ul of DMEM medium were injected directly into their fourth mammary fat pat (MFP). SH was delivered intraperitoneally daily, beginning the day after 4T1 cells injection. For early-stage primary tumor study, single-cell suspensions of 2 × 10^5^ 168FARN cells were injected directly into their fourth mammary fat pat. Body weight and tumor volume (V = 0.5236 × a × b^2^, where a is the major tumor axis and b is the minor tumor axis) were measured every two days. The mice were sacrificed at defined time intervals after cell inoculation. Tumor, lungs and livers were collected for histological analysis.

To examine the lung metastasis foci, tissues were fixed in Bouin's solution for 2 days and then observed under the anatomic microscope (Olympus SZ61, Japan). The liver metastasis was evaluated by counting the metastatic foci in five randomly selected fields by biological microscope (Olympus BX51, Japan) in each specimen of liver H&E staining.

For experimental metastasis assay, 1 × 10^6^ 4T1 cells were injected into the tail vein, and treatment commenced 12 hours later. Lung tissue was collected after fixation by intratracheal instillation of 4% paraformaldehyde after 9 days treatment. Three representative 5-μm sections (at least 100 μm apart) through one lobe of each lung were stained with hematoxylin and eosin. Each tumor focus was graded as intra- or extravascular.

### Immunohistochemistry, immunofluorescence and western blot

Immunohistochemistry of CD31 (1:100, Abcam, UK), PCNA (1:400, Santa Cruz, USA) and HIF-1α (1:800, Bioss, China) were performed in paraffin sections (3 μm). IF double-staining of CD31 (1:50) and α-SMA (1:100, Boster, China) was done in frozen sections. Sections were then incubated with the appropriate fluorescently conjugated secondary antibodies or with peroxidase-labeled IgGs. Stainings for CD206 (1:100, AbD Serotec, UK), iNOS (1:50, Santa Cruz, USA), F4/80 (1:100, AbD Serotec, UK), CD11b (1:100, AbD Serotec, UK) and Gr-1 (1:100, AbD Serotec, UK) were done by using directly conjugated antibodies. Sections were then observed under camera attached microscope (Olympus BH2, Japan) or laser confocal microscope (Leica SP5 II, Germany). Protein level of HIF-1α in the tumor lysate was also determined by immunoblot analysis and the primary antibody of HIF-1α (1:200, Bioss, China) and β-actin (1:5000, Proteintech, China) were used.

### Vessel perfusion assay

Vessel perfusion was detected by intravenous injection of 0.05 mg FITC-labelled lectin in tumor-bearing mice. Ten minutes later, mice were perfused by intracardiac injection of saline (5 min) and 2% PFA (7 min). Tumors were then harvested and frozen in OCT medium. Tumor perfusion was analyzed by counting the number of FITC-lectin (Vector Laboratories, USA) and CD31 positive vessels.

### Mouse angiogenesis antibody array

Four randomly selected tumor lysates from each group were applied to a Mouse Angiogenesis Antibody Array (RayBiotech, USA) to analyze the expression of angiogenesis-related molecules according to the manufacturer's protocol. Duplicate dots identifying each protein were scanned and quantified by Genepix 4000B Microarray Scanner.

### ELISA assay for serum, tumor extracellular fluid and cell lysate

Snap-frozen tumor samples were crushed into fragments <2 mm in diameter and resuspended with 1:2 weight/volume of 2×PBS and rotated at 4°C for 2 h. The samples were vortexed then spun for 3 min at 5,000 RPM then the tumor extracellular fluid was collected^36^. Collected cultured cells and diluted to 2 × 10^6^/ml with PBS. Broke up cells by ultrasonic and spun for 10 min at 10000 g then the cell lysate was collected. Serum samples and/or tumor extracellular fluid and/or cell lysate from the control and SH-treated groups were applied to ELISA kits, following the manufacturer 's protocol to quantify bFGF (CUSABIO, China), PF-4 (CUSABIO, China), G-CSF (RayBiotech, USA) and GM-CSF (RayBiotech, USA).

### Statistical analysis

Quantitative variables were expressed as mean ± standard deviations and analyzed by *t-*test or one-way ANOVA or two-way ANOVA. The intravascular and extravascular growth of metastatic tumor cells was analyzed by Chi-square test. All statistical tests were two-sided and *p* < 0.05 was considered to statistically significant.

### Ethics statement

The methods were carried out in accordance with the approved guidelines. Animal care and experimental protocol were approved by the Experimental Animal Ethics Committee of Xi'an Jiaotong University.

## Author Contributions

H.Z. and J.H. designed the research and wrote the paper; H.Z., L.Z. and X.L. conducted the *in vitro* experiments; H.Z., X.T. and Y.L. performed the *in vivo* study; Y.R. and K.W. analyzed data; P.L. and C.Z. participated in the experimental design. All authors have read and approved the manuscript.

## Supplementary Material

Supplementary Information Supplementary data
