# Supplementary material for: Vascular Normalization Induced by Sinomenine Hydrochloride Results in Suppressed Mammary Tumor Growth and Metastasis
Source: Sci Rep. 2015 Mar 9;5:8888. doi: 10.1038/srep08888 (PMC4352869; doi:10.1038/srep08888)
Supplement: Supplementary Information — Supplementary data [file srep08888-s1.pdf]

Vascular Normalization Induced by Sinomenine Hydrochloride Results in Suppressed Mammary Tumor Growth and Metastasis

Huimin Zhang[1], Yu Ren[1], Xiaojiang Tang[1], Ke Wang[1], Yang Liu[1], Li Zhang[1], Xiao Li[1], Peijun Liu[2], Changqi Zhao[3], Jianjun He[1,*]

[1]Department of Surgical Oncology, The First Affiliated Hospital, Xi'an Jiaotong University College of Medicine, 277W. Yanta Road, Xi'an, Shaanxi 710061, P.R. China.

[2]Translational Medical center, First Affiliated Hospital of Xi'an Jiaotong University, 277 West Yanta Road, Xi’an 710061, P.R. China.

[3]Key Laboratory of Cell Proliferation and Regulation Biology, College of Life Science, Beijing Normal University, Beijing 100875, P.R. China.

[*]corresponding author: Jianjun He, Department of Surgical Oncology, The First Affiliated Hospital, Xi'an Jiaotong University College of Medicine, 277 West Yanta Road, Xi'an, Shaanxi 710061, P. R. China. Tel and Fax: +86 29 85324609. E-mail: chinahjj@163.com

Supplementary Figures S1-S4

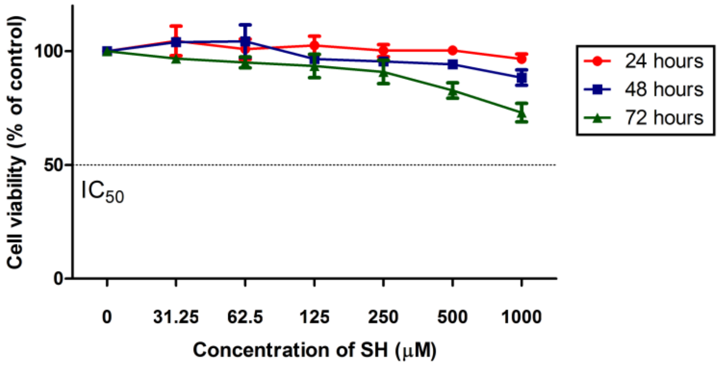

Fig. S1 Cytotoxic effect of SH on HUVECs. Cytotoxicity on endothelial cells by SH is investigated by MTT assay. Cell viability (%) = $OD_{treatment\ group}$ / $OD_{control\ group}$ × 100%. N=3.

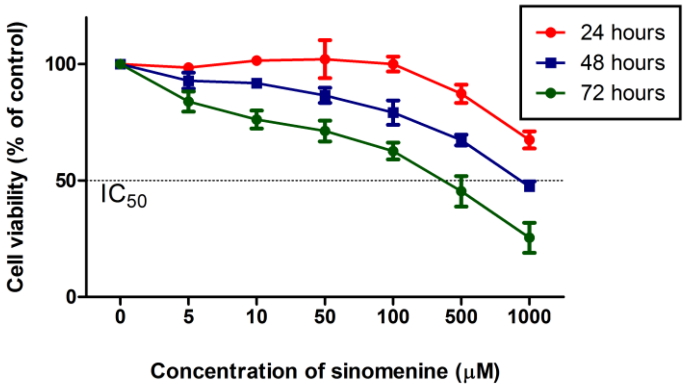

Fig. S2 Effect of SH on 4T1 cells proliferation in vitro. The median inhibitory concentration of SH on 4T1 cells is determined by MTT assay. Cell viability (%) = $OD_{treatment\ group}$ / $OD_{control\ group}$ ×100%. N=3.

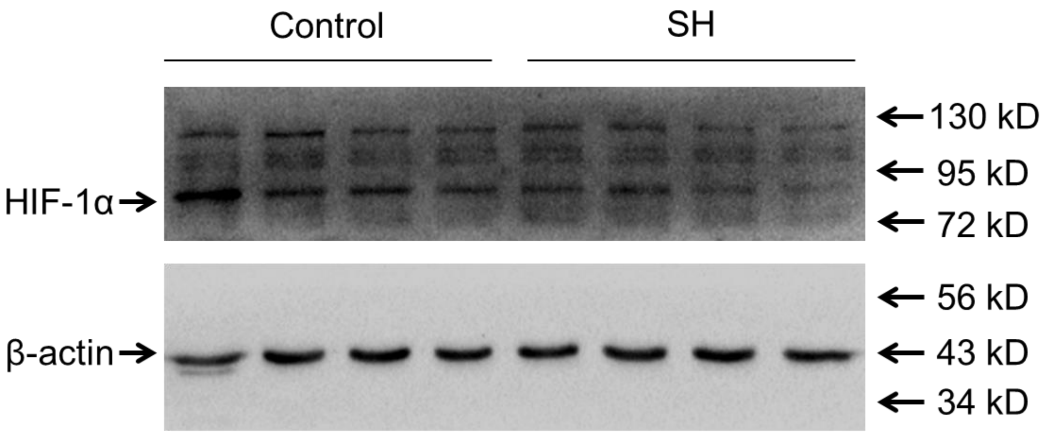

Fig. S3 SH inhibits accumulation of HIF-1α in tumors. Tumor lysates were prepared and a western analysis for the indicated proteins was performed. Each sample has the expression of β-actin as internal control. Full-length blots are shown.

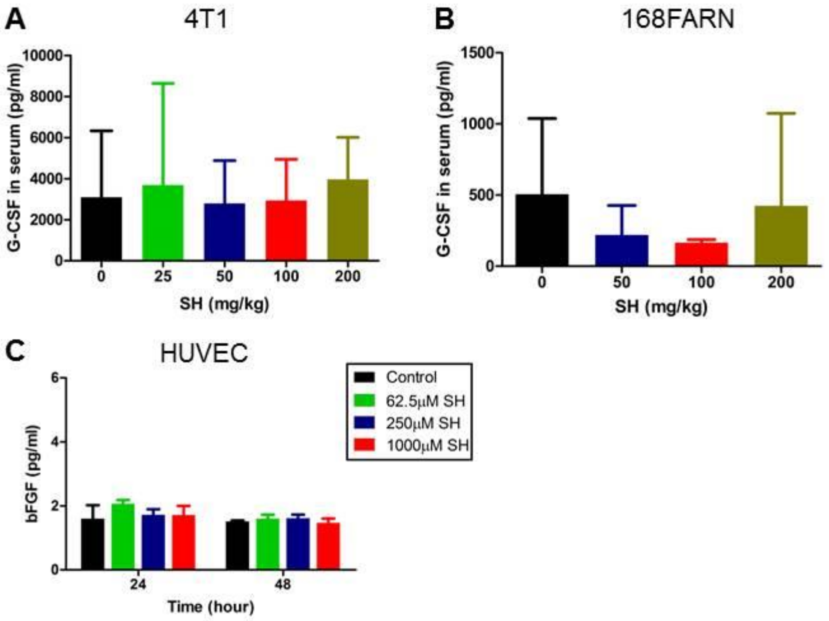

Fig. S4 Effect of SH on angiogenic factors. (A, B) G-CSF levels in mice serum were measured by ELISA assay. N=6. **(C)** bFGF expressions in HUVECs were determined by ELISA assay. N=3.

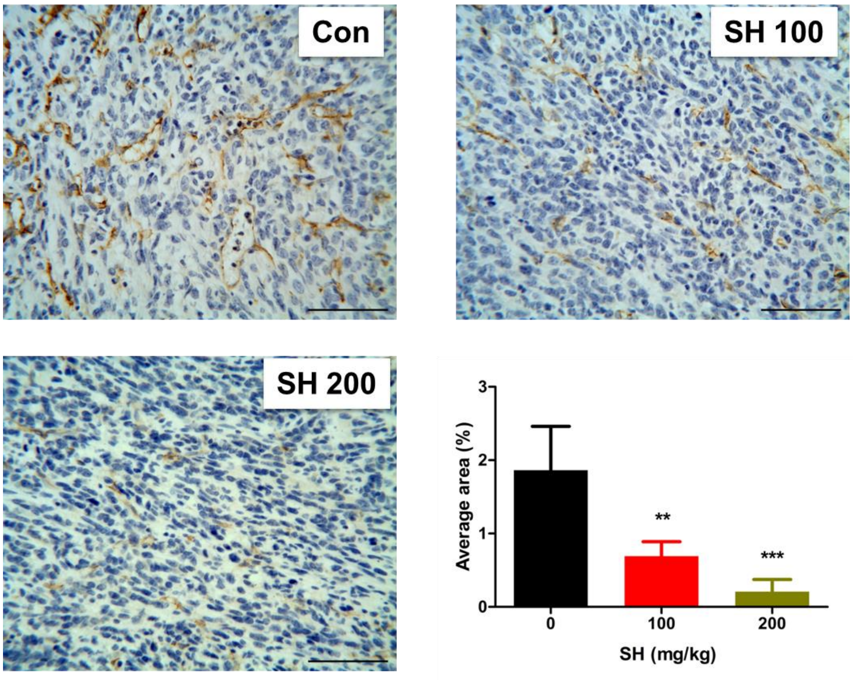

Fig. S5 Antiangiogenic effect of SH in vivo. IHC staining of EC marker CD31 and vessel areas were qualified in the column graph. Bar: 100μm. Statistical significance: P < 0.01 (**) or < 0.001 (***), N=6.
